# Gammaherpesvirus Readthrough Transcription Generates a Long Non-Coding RNA That Is Regulated by Antisense miRNAs and Correlates with Enhanced Lytic Replication In Vivo

**DOI:** 10.3390/ncrna5010006

**Published:** 2019-01-10

**Authors:** Mehmet Kara, Tina O’Grady, Emily R. Feldman, April Feswick, Yiping Wang, Erik K. Flemington, Scott A. Tibbetts

**Affiliations:** 1 Department of Molecular Genetics & Microbiology, UF Health Cancer Center, University of Florida, Gainesville, FL 32610, USA; mhmtt.kara@gmail.com (M.K.); emy.feldman@gmail.com (E.R.F.); feswicka@ufl.edu (A.F.); yipingwang@ufl.edu (Y.W.); 2 Laboratory of Protein Signaling and Interactions, GIGA-R (MBD), University of Liège, 4000 Liège, Belgium; christina.ogrady@ulg.ac.be; 3 Department of Pathology, Tulane Cancer Center, Tulane University, New Orleans, LA 70112, USA; erik@tulane.edu

**Keywords:** non-coding RNA, lncRNA, virus, gammaherpesvirus, MHV68

## Abstract

Gammaherpesviruses, including the human pathogens Epstein–Barr virus (EBV) and Kaposi’s sarcoma-associated herpesvirus (KSHV) are oncogenic viruses that establish lifelong infections in hosts and are associated with the development of lymphoproliferative diseases and lymphomas. Recent studies have shown that the majority of the mammalian genome is transcribed and gives rise to numerous long non-coding RNAs (lncRNAs). Likewise, the large double-stranded DNA virus genomes of herpesviruses undergo pervasive transcription, including the expression of many as yet uncharacterized lncRNAs. Murine gammaperherpesvirus 68 (MHV68, MuHV-4, γHV68) is a natural pathogen of rodents, and is genetically and pathogenically related to EBV and KSHV, providing a highly tractable model for studies of gammaherpesvirus biology and pathogenesis. Through the integrated use of parallel data sets from multiple sequencing platforms, we previously resolved transcripts throughout the MHV68 genome, including at least 144 novel transcript isoforms. Here, we sought to molecularly validate novel transcripts identified within the *M3/M2* locus, which harbors genes that code for the chemokine binding protein M3, the latency B cell signaling protein M2, and 10 microRNAs (miRNAs). Using strand-specific northern blots, we validated the presence of *M3-04,* a 3.91 kb polyadenylated transcript that initiates at the M3 transcription start site and reads through the M3 open reading frame (ORF), the M3 poly(a) signal sequence, and the M2 ORF. This unexpected transcript was solely localized to the nucleus, strongly suggesting that it is not translated and instead may function as a lncRNA. Use of an MHV68 mutant lacking two *M3-04*-antisense pre-miRNA stem loops resulted in highly increased expression of *M3-04* and increased virus replication in the lungs of infected mice, demonstrating a key role for these RNAs in regulation of lytic infection. Together these findings suggest the possibility of a tripartite regulatory relationship between the lncRNA *M3-04*, antisense miRNAs, and the latency gene *M2*.

## 1. Introduction

For years, our understanding and interpretation of mammalian genomes was almost exclusively focused on the 1% of the genome that is protein-coding. However, over the last 15 years, it has become clear that, for a wide range of organisms, many abundant and diverse RNA molecules are transcribed from previously unannotated regions of the genome [1]. Moreover, it is now apparent that herpesviruses, as entities that co-evolve with their hosts, also occupy this transcriptional realm to promote their life cycles. In recent years, studies have shown that several herpesviruses undergo pervasive transcription, revealing novel coding and noncoding RNAs. For example, deep sequencing analysis of human cytomegalovirus (HCMV)-infected fibroblasts revealed that approximately 65% of virus-encoded, polyadenylated transcription is committed to four non-coding RNAs: *RNA2.7*, *RNA1.2*, *RNA4.9*, and *RNA5.0* [2]. 

Such non-coding RNAs can play functional roles in virus infection. For example, *RNA2.7* inhibits interferon-induced apoptosis and protects infected cells from complex I-driven ATP production blocking [3]. Likewise, the human gammaherpesvirus Kaposi’s sarcoma-associated herpesvirus (KSHV) expresses at least two long non-coding RNA (lncRNA) that are likely important players in specific phases of infection: the 1.08 kb polyadenylated nuclear (*PAN*) RNA accumulates to high levels during lytic infection [4,5,6,7], and the 10 kb antisense-to-latency transcript (*ALT*) lies antisense to the KSHV latency associated region [8,9]. Further, ribosome profiling and RNA-seq analyses of KSHV-infected epithelial cells revealed highly pervasive transcription from the KSHV genome, including several antisense transcripts that function in part as non-coding RNAs [10]. Similar widespread intergenic transcription and latency-region antisense transcripts have been observed from the alphaherpesvirus herpes simplex virus (HSV)-1 genome [11]. 

Despite these advances, globally resolving specific overlapping transcripts within these dense herpesvirus genomes has been extraordinarily difficult. Recently though, O’Grady et al. have overcome this obstacle for the human gammaherpesvirus Epstein-Barr virus (EBV) through integration of parallel data sets from multiple genomics platforms. While PacBio SMRT sequencing facilitates the identification of long-read transcript isoforms, individual full-length transcript isoforms can be validated by bioinformatically coupling parallel data sets from Illumina RNA-seq and deepCAGE platforms using their novel pipeline TRIMD (transcript resolution through integration of multi-platform data). This approach revealed almost 300 novel polyadenylated EBV transcripts, which were generated from complex promoter usage, intergenic transcription, alternative splicing, and readthrough transcription [12].

Murine gammaherpesvirus 68 (MHV68, MuHV-4, γHV68) is a natural pathogen of rodents that is genetically and pathogenically related to the human gammaherpesviruses EBV and KSHV. Like the human viruses, MHV68 establishes lifelong latent infection in B cells and is associated with the development of lymphoproliferative diseases and lymphoma. Although the MHV68 genome was first published more than 20 years ago [13], the annotation of the genome has remained primarily confined to open reading frames (ORFs). However, later studies used high density tiling microarrays to demonstrate a higher level of complexity of the MHV68 transcriptome, revealing 30 areas of contiguous transcriptional activity, called expressed genomic regions (EGRs), which are interspersed throughout the genome [14,15]. Nevertheless, the identification of specific transcripts emanating from these regions has remained elusive. Very recently, we applied the TRIMD approach to MHV68 to globally resolve full-length MHV68 transcript isoforms during lytic infection. Through the coupling of PacBio long-read sequencing, with parallel data sets from Illumina RNA-seq and deepCAGE platforms, we resolved 258 MHV68 transcript isoforms, including transcripts for 55 truncated, spliced, or novel ORFs, and up to 25 potential non-coding RNAs [16]. One region of particular interest that emerged from these studies was the *M3/M2* locus, which encodes (i) the important chemokine binding protein M3, (ii) the critical latency-associated B cell signaling protein M2, and (iii) the *TMER 6*, *7*, and *8* genes, which encode up to 10 microRNAs (miRNAs). In work described here, we validated the presence of an intriguing and unexpected transcript, *M3-04*, which overlaps both of the key genes in this region. We demonstrated that *M3-04* is polyadenylated and localized to the nucleus, strongly suggesting that it functions as a lncRNA. We have further defined suppression of *M3-04* transcription by antisense miRNAs, suggesting a tight regulatory relationship between *M3-04*, antisense miRNAs, and the overlapping latency gene *M2*. 

## 2. Materials and Methods

### 2.1. Cell Lines and Viruses

The murine fibroblast cell line NIH 3T12 was maintained in Dulbecco’s modified Eagle’s medium (DMEM) with 10% fetal calf serum, 100 U/mL of penicillin, 100 mg/mL streptomycin, and 2 mM l-glutamine. The murine B cell lines, A20, HE2.1, and WEHI 231 cells were maintained in complete RPMI 1640 with 10% fetal calf serum, 100 U/mL penicillin, 100 mg/mL streptomycin, 2 mM l-glutamine, and 50 µM β-mercaptoethanol. HE2.1 cells were maintained in 300 µg/mL hygromycin. Reactivation of the virus was induced using 20 ng/mL 12-*O*-tetradecanoylphorbol-13-acetate (TPA). Unless otherwise specified, MHV68.OR73Bla, a recombinant virus which expresses β-lactamase as a fusion to the LANA protein encoded by *ORF73*, was used for the assays. Virus stocks were prepared and titered using a plaque assay on NIH 3T12 fibroblasts.

### 2.2. RNA Extractions and Northern Blots

RNA extraction and northern blot protocol is mainly based on the protocol by McClure et al. [17]. Briefly, 2 × 10^6^ NIH 3T12 fibroblasts were infected at multiplicity of infection (MOI) 5 with MHV68, and at 24 hpi cells were harvested, lysed in Trizol, and RNA was extracted according to the manufacturer’s protocol [17]. The northern blot protocol for longer RNA molecules (>200) was as follows: 5 μg total RNA sample was loaded onto a 6% formaldehyde-containing 1% agarose gel RNA Millenium Marker (Ambion, Thermo Fisher Scientific, Waltham, MA, USA). The gel was run in a 3-(*N*-morpholino)propanesulfonic acid (MOPS) buffer, then blotted onto a Hybond XL nylon membrane (Life Technologies, Thermo Fisher Scientific, Waltham, MA, USA) overnight with Turbolotter kit in 20X SSC buffer. The membrane was washed, RNA was crosslinked to the membrane using UV light (Spectrolinker XL1500 UV Crosslinker, Spectronics Corporation, Westbury, NY, USA), and the membrane was stained with 0.02% methylene blue for visualization of the RNA integrity and markers. The crosslinked membrane was prehybridized at 63 °C for 4 hours in an ULTRAhyb (Ambion) buffer and then labeled probe (see below) was added for overnight incubation. The next day the membrane was washed three times with a 1x SSC buffer and exposed to a film at 80 °C for appropriate time.

### 2.3. Probe Generation

Riboprobes were prepared with Maxiscript T7/Sp6 (Thermo Fisher Scientific, Waltham, MA, USA). Briefly, the probe template was PCR amplified with primers containing T7 and Sp6 promoters on each side. The template was labeled by adding 10 µCi α-CTP (Perkin Elmer, Waltham, MA, USA) to the reaction for four hours at 37°C. The DNA template was digested with DNase for 20 minutes, then the reaction was stopped by adding EDTA. The probe was used without purification unless specified. For end labeling, 10 nM of the antisense oligo (IDT) was labeled with [γ-32P]-ATP (Perkin Elmer) using T4 PNK at 37 °C for one hour. Primers used for the probe templates are listed in Table S1.

### 2.4. Nuclear and Cytoplasmic Fractionation for RNA Extraction

NIH 3T12 fibroblast cells were infected at MOI 5 with MHV68. At 16 hpi, infected cells were washed, trypsinized, and then collected and subjected to 500× *g* centrifugation (Beckman Coulter GS-6R Centrifuge, Brea, CA, USA) for 5 minutes at 4 °C. Cells were resuspended in 400 µL of Buffer 1 (0.32 M Sucrose, 3 mM CaCl_2_, 2 mM MgCl_2_, 0.1 mM EDTA, 10 mM Tris pH8, 0.5 % Igepal (Nonidet P40), 1 mM DTT 0.4U/µL RNasin). Cells were then incubated on ice for 10 minutes and centrifuged at 500× *g* for 5 minutes at 4 °C. The supernatant fluid was transferred to a new microcentrifuge tube and equal volume of Trizol added. The pellet was resuspended in 400 µL of Buffer 2 (150 mM NaCl, 50 mM, 5 mM, 0.1 % Triton, 0.1 % SDS) and an equal volume of Trizol was added to the samples. The RNA was then extracted according to the manufacturer’s protocol.

### 2.5. Poly(A)^+^ RNA Selection

Poly(A)Purist MAG Kit (Ambion) was used to extract poly(A)^+^ RNA, according to the manufacturer’s recommendation. Briefly, total RNA was incubated with OligodT Magbeads in 1x binding solution at room temperature for an hour, captured with a magnetic stand, then washed twice with a wash buffer and eluted twice with an elution buffer in 200 µL each. The eluate was then ethanol precipitated with 40 µL of 5 M ammonium acetate, 1 µL glycogen, and 1.1 mL 100% ethanol, then centrifuged at 16,000× *g* for 30 minutes at 4 °C, washed with 70% ethanol again, and finally resuspended in DEPC (diethyl pyrocarbonate) H_2_O.

### 2.6. Mutant Virus Generation

The MHV68Δ15.9 mutant virus was generated via deletion of the *mghv-miR-M1-15* and *-9* pre-miRNA stem-loops, but leaving the pol III promoter, full viral tRNA-like element, pol III stop sequence, and intervening sequences intact (Table S2). Mutations were generated in the context of a wild-type bacterial artificial chromosome (BAC)-derived MHV68 that expresses a β-lactamase marker for tracking infected cells in vivo [18]. Parental wild-type MHV68 BAC [19] and MHV68.ORF73βla BAC [18] have been previously described. The mutant virus was generated in three stages using two-step Red-mediated recombination [20] onto a wild-type MHV68.ORF73βla BAC backbone. Briefly, PAGE-purified primers with sequence homologous to both the viral sequence flanking the desired mutation site and the *kanamycin* (*Kan*) resistance gene were used to amplify the *Kan* gene. The forward primer, including the *Kan* sequence, also contained an I-SceI homing endonuclease cutting site. The resulting amplicon was purified and electroporated into GST1783 *Escherichia coli* cells containing the MHV68.ORF73βla BAC backbone and Red recombinase machinery. Transformed cells were recovered and grown at 30 °C overnight on *Kan* selection medium. DNA extracted from the resulting colonies was isolated and digested with XhoI and then analyzed by pulse-field gel electrophoresis (PFGE) to screen for primer insertion and to confirm genomic integrity. Positive clones were subjected to an I-SceI-mediated recombination, which was induced using 1% arabinose. Mutants were validated using PFGE, PCR, and sequencing. BAC DNA from a single positive clone was then isolated and transfected into NIH 3T12 cells by using a TransIT-3T3 transfection kit (Mirus Bio, Madison, WI, USA). The resulting virus was passaged twice and amplified on Cre recombinase-expressing NIH 3T12 cells to remove the BAC cassette. Titers of final viral BAC-minus stocks were determined by plaque assay on NIH 3T12 cells. qRT-PCR was performed on adjacent genes using primers listed in Table S1. 

### 2.7. Mouse Infections

Female C57BL6/J (B6) mice were purchased from Jackson Laboratory (Bar Harbor, ME, USA) at 7 to 8 weeks and were housed in a biosafety level 2+ (BSL2+) facility at the University of Florida, Gainesville, FL, USA, in accordance with all federal guidelines and as approved by the University of Florida Institutional Animal Care & Use Committee (protocol #201609615, 12 September 2018). For all infections, eight mice per sample group were anesthetized with isoflurane and then inoculated intranasally (i.n.) with 10^4^ PFU virus in 30 μL serum-free DMEM. Lungs were harvested at 5 days post-infection. 

### 2.8. Plaque Assays

To determine the titer of viral stocks, samples were prepared as 10-fold dilutions in serum-free DMEM, added to a single well of a six-well plate containing a monolayer of 2 × 10^5^ NIH 3T12 fibroblasts, and then overlaid with 1:1 mixture of methylcellulose (Sigma, St Louis, MO, USA) and 2x MEM Temin’s modification, no phenol red medium supplemented with 5% fetal calf serum, 100 U/mL of penicillin, and 100 mg/mL streptomycin. After 7 days, neutral red stain was added to the assays to visualize and count plaques. To determine in vivo virus titers, lungs were harvested from four mice per sample group per experiment. Plaque assays were then performed as previously described [21]. Briefly, harvested lung tissues were placed in sterile 2 mL screw cap tubes containing 1 ml of DMEM and 500 μL of 1 mm zirconia-silica beads (BioSpec Products, Bartlesville, OK, USA) and stored at −80 °C until use. Samples were thawed on ice and tissues were homogenized using a Mini-BeadBeater (BioSpec Products). Samples were serially ten-fold diluted in complete DMEM, and plaque assays performed as described for virus stocks. 

## 3. Results

### 3.1. M3-04 Is a Novel Readthrough Transcript Spanning the M3 and M2 Open Reading Frames

**Figure 1:**
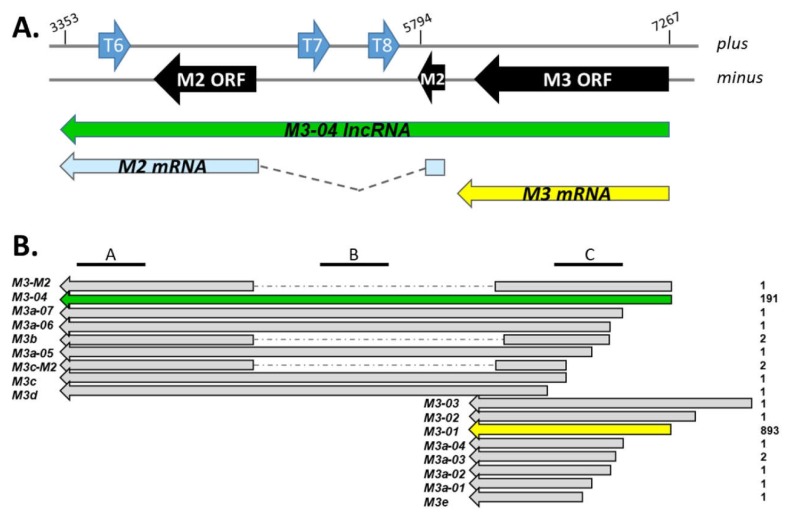
Map of genes and transcripts within the M3/M2 locus. (**A**) The *M3/M2* locus across plus and minus strands of the MHV68 genome. ORFs are indicated in black, *TMER* genes are indicated in blue. Below the genome, the major RNA transcripts within the locus are indicated, including *M3-04* (nt positions 7267 to 3353 within NC_001826.2). (**B**) *M3/M2* locus transcripts identified through TRIMD analysis of multi-platform transcriptomics data sets. Transcript names are indicated at left, TRIMD scores for each transcript are indicated on the right. Location of northern blot probes A, B, and C are indicated at top.

Interestingly, TRIMD analysis (Figure 1) revealed the 17 transcript isoforms that contain at least some portion of the M3 open reading frame (ORF). In addition to the expected short transcripts encoding full-length M3 (*M3-01, M3-02, M3-03*), TRIMD analysis identified ten transcripts encoding different 5’ truncated versions of M3 (*M3a, M3c, M3d*, and *M3e* isoforms), one isoform of M3 containing a splice to a downstream exon (*M3b-01*), and two isoforms encoding full-length or 5’ truncated versions of M3 spliced to the downstream M2 ORF (*M3-M2, M3c-M2*). 

All of these transcripts have been bioinformatically validated across the three transcriptomics platforms; however, their biological importance is likely to be at least partially linked to level of expression. Although PacBio Iso-Seq score, which reflects PacBio coverage of consensus full-length transcript isoforms, is non-quantitative, the score can provide some degree of insight into a relative level of expression within a given sample. Thus, it is notable that within this locus, two transcript isoforms stand out. In stark contrast to the other 15 isoforms, which have scores of 1 or 2, the score for *M3-01* was 893, and the score for *M3-04* was 191. The high score for *M3-01* is consistent with its likely function as the primary transcript for the highly expressed, full-length M3 protein. Unexpectedly though, the *M3-04* transcript initiates from the same transcription start site as the major M3 transcript *M3-01*, but appears to originate from readthrough of the *M3-01* polyadenylation signal sequence. This novel 3.91 kb isoform is of particular interest because it: (i) fully overlaps the gene that encodes the important chemokine binding protein M3, (ii) fully overlaps the gene that encodes the critical latency-associated B cell signaling protein M2, and (iii) runs antisense to and fully overlaps the *TMER 6*, *7*, and *8* genes, which encode up to 10 miRNAs. 

**Figure 2:**
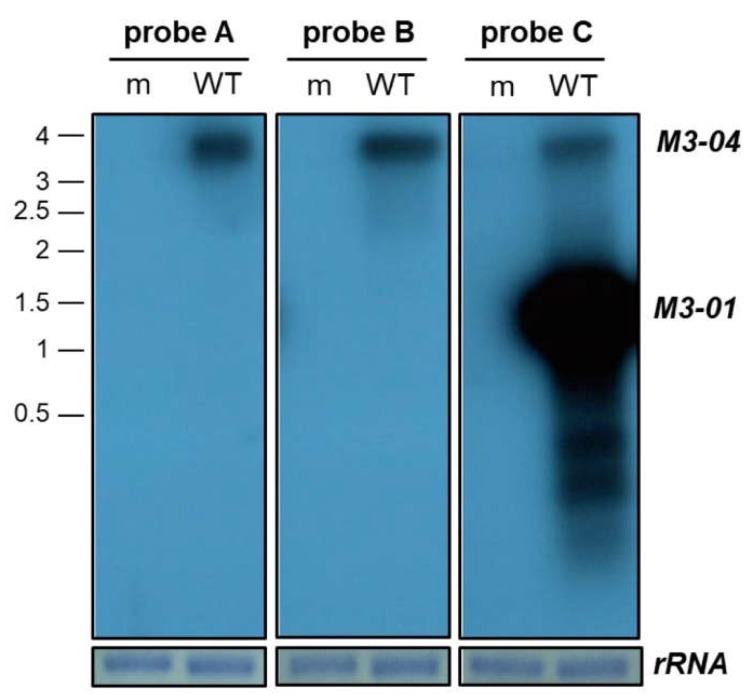
Northern blot validation of *M3-04* transcript. RNA was harvested from NIH 3T12 fibroblasts at 18 hours from cells that were mock-infected (m), or infected with wild-type MHV68 (WT) at MOI 5. Three different strand-specific probes were used on northern blots to detect transcripts expressed within the *M3/M2* locus. Probes A, B, and C detected an approximately 3.9 kb transcript, corresponding to *M3-04*. Probe C also detected an approximately 1.6 kb transcript, corresponding to *M3-01*, as well as two smaller RNAs. An 18S ribosomal RNA (rRNA) on the methylene blue stained membrane is shown as the loading control.

To molecularly validate the *M3-04* transcript during lytic MHV68 infection, we initially performed northern blot analysis using a series of probes across the *M3/M2* locus (Figure 2). Following mock or MHV68 infection of NIH 3T12 fibroblasts, total RNA was isolated and subjected to formaldehyde gel electrophoresis. Following transfer, membranes were hybridized with probes that lie either within the M3 open reading frame (probe C), within the M2 intron (probe B), or within the M2 3’ untranslated region (3’ UTR) (probe A). As expected, probe C identified an intense band at approximately 1.6 kb, consistent with the size of the *M3-01* isoform, which is very likely the primary transcript that codes for the highly expressed M3 protein. Probe C also identified at least two additional isoforms of less than 1 kb, which likely represent the 5’ truncated isoforms of M3. Probe C also identified a transcript of approximately 3.9 kb. Notably, this band corresponded to the size of the only transcript apparent in blots hybridized with the downstream probes A or B. Thus, these findings demonstrate the presence of a 3.9 kb transcript that spans from upstream of the M3 ORF to the M2 3’ UTR, validating the existence of the novel *M3-04* isoform.

**Figure 3:**
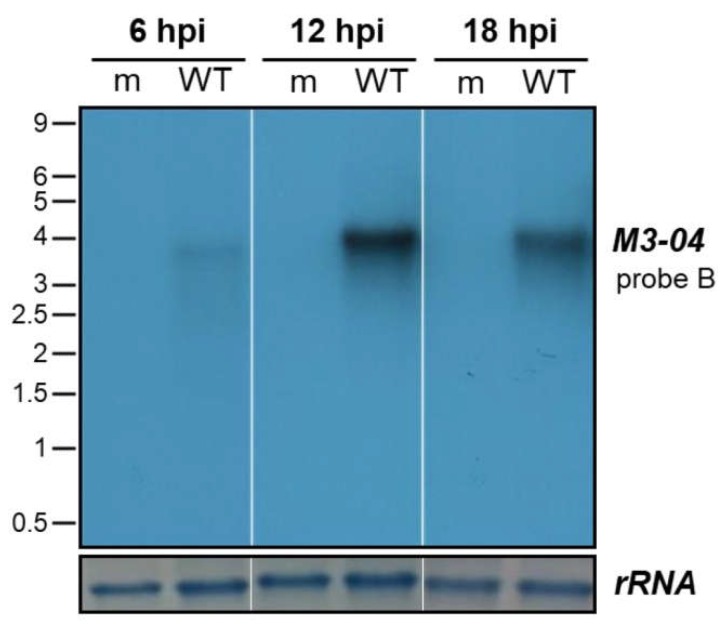
Timecourse of *M3-04* expression. NIH 3T12 fibroblasts were mock-infected (m) or infected with wild-type MHV68 (WT) at MOI 5. At 6, 12, and 18 hpi, total RNA was harvested and northern blot analysis was performed using probe B to detect *M3-04* transcript. 18S rRNA on the methylene blue stained membrane is shown as the loading control.

To determine the kinetics of *M3-04* expression, we infected NIH 3T12 fibroblasts with MHV68 at MOI 5, and harvested RNA samples at 6-hour intervals throughout a full course of lytic infection (Figure 3). Although *M3-04* expression was detectable at low levels as early as 6 hpi, transcript levels appeared to peak at 12 hpi, with stable expression detectable through 18 hpi. These findings further validate *M3-04* as a bona fide MHV68 transcript expressed throughout lytic replication.

### 3.2. M3-04 Is a Polyadenylated, Nuclear Transcript

**Figure 4:**
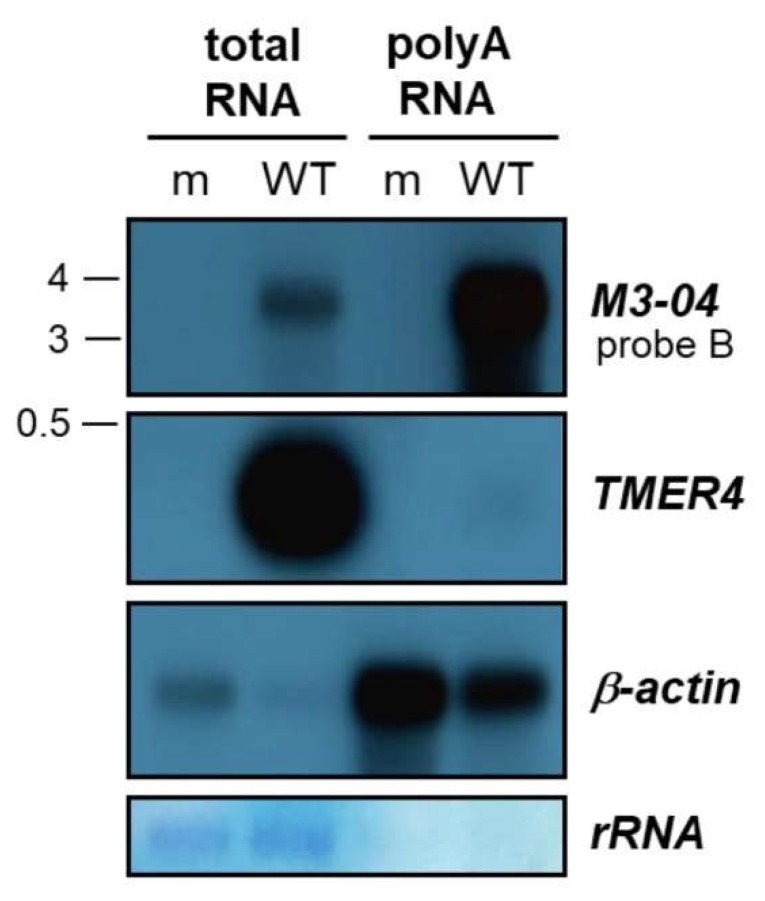
*M3-04* is polyadenylated. Poly(A)-selected or total RNA was harvested at 18 hours from either mock-infected (m) or wild-type MHV68-infected (WT) NIH 3T12 fibroblasts. Northern blot was performed using probe B to detect *M3-04* transcript. *TMER4* and *β-actin* transcripts were detected using strand-specific probes. Methylene blue staining is shown for rRNA as a loading and poly(A) selection control.

To determine the polyadenylation state of the *M3-04* transcript, we isolated total and poly(A) RNA from mock- and MHV68-infected NIH 3T12 fibroblasts. RNAs were then resolved on formaldehyde gels and northern blotting was performed using probe B (which lies downstream of *M3* and within the *M2* intron) to detect *M3-04,* or control probes to detect the non-polyadenylated, pol III-derived MHV68 transcript *TMER4* or the polyadenylated host transcript *β-actin* (Figure 4). As expected, *TMER4* was abundantly present in total RNA but absent from poly(A)-enriched RNA. In contrast, both MHV68 *M3-04* and host *β-actin* were present in moderate amounts in total RNA, but highly enriched in poly(A) RNA.

**Figure 5:**
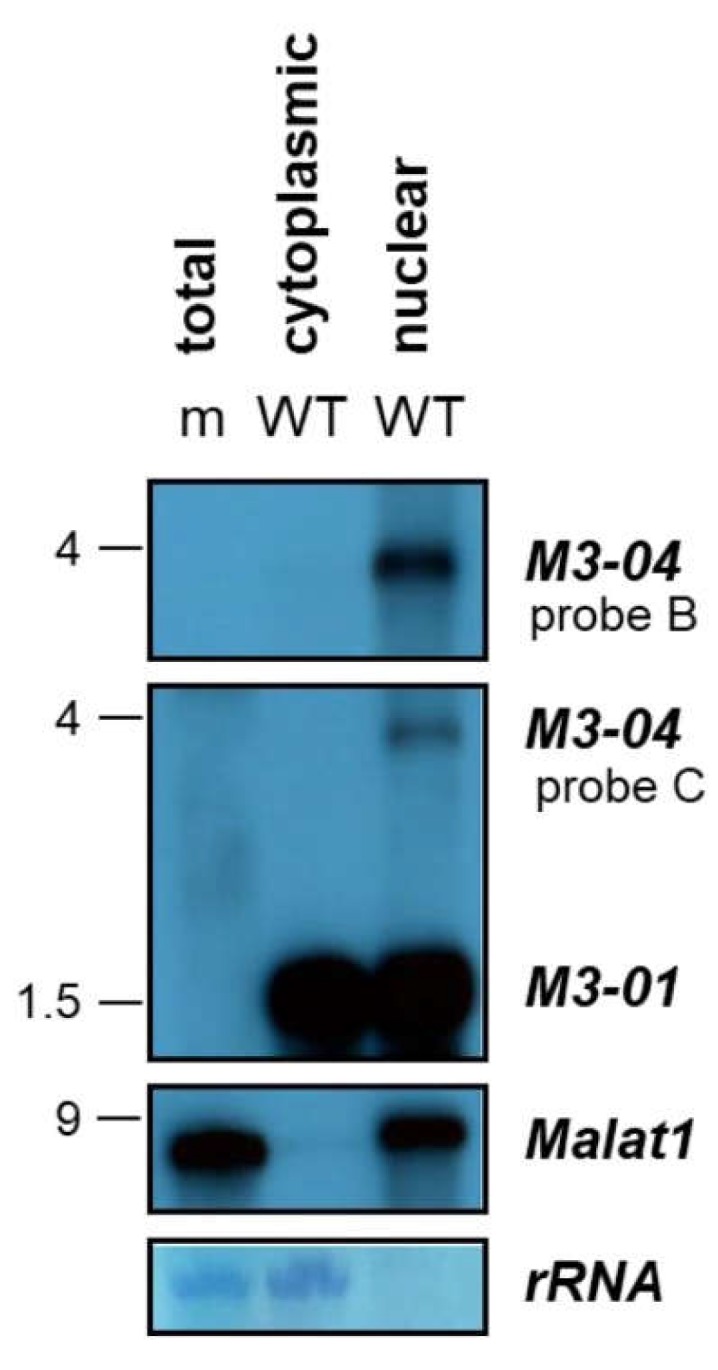
*M3-04* is a nuclear transcript. Total RNA was harvested at 16 hours from mock-infected (m) or wild-type MHV68-infected (WT) NIH 3T12 fibroblasts, and cytoplasm and nuclear fractions were obtained. Northern blots were probed with strand-specific *M3/M2* locus probes B or C, or with probe to the control nuclear host RNA *Malat1*. Methylene blue staining is shown for rRNA as a loading control.

To determine the subcellular localization of *M3-04*, we harvested total RNA from MHV68-infected NIH 3T12 fibroblasts and fractionated nuclear and cytoplasmic total RNA. Total RNA from mock-infected (m) cells was included as a virus transcript probe specificity control. Northern blots were hybridized with probe B to detect *M3-04*, probe C to detect both *M3-01* and *M3-04*, or a probe to the nuclear host lncRNA *Malat1* (Figure 5). While *M3-01*, the primary transcript encoding M3 protein, was detected in abundance in both the cytoplasmic and nuclear RNA fractions, while the novel transcript *M3-04* was only detected in the nuclear fraction of infected cells. Similarly, the well-characterized lncRNA *Malat1*, which is exclusively nuclear, was detected here only in the nuclear RNA fraction. Thus, together these data clearly demonstrate that *M3-04* is a polyadenylated, nuclear transcript. Moreover, because polyadenylation and nuclear localization are very common features of noncoding RNAs, and because most nuclear transcripts are not translated to proteins, these findings strongly implicate *M3-04* as a novel, MHV68-encoded lncRNA.

### 3.3. The M3-04 lncRNA Is Expressed During Lytic But Not Detectable During Latent Infection

**Figure 6:**
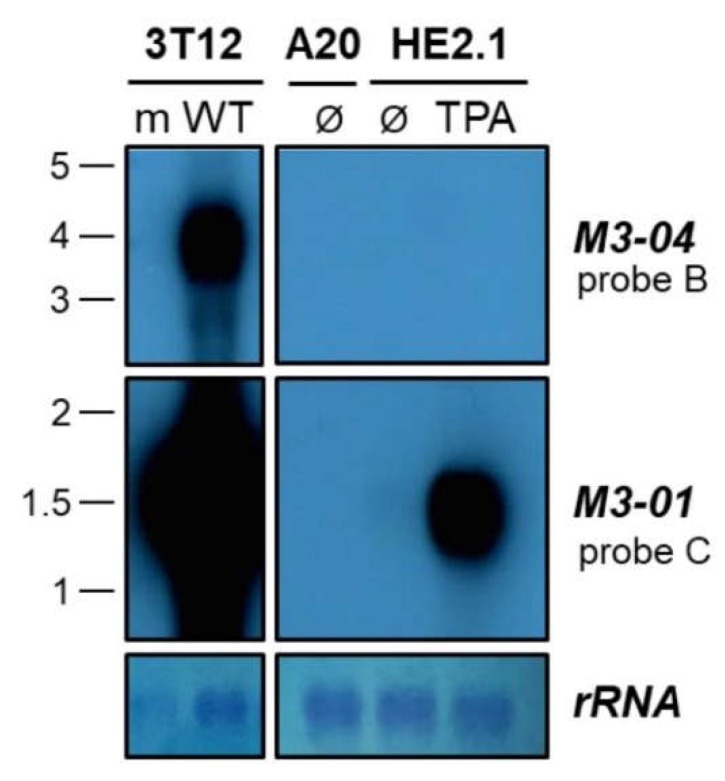
*M3-04* is expressed during lytic replication in fibroblasts, but not in latent or reactivated B cells. Total RNA was harvested at 18 hours from mock-infected (m) or wild-type MHV68-infected (WT) NIH 3T12 fibroblasts, or from B cells lines A20 (uninfected), HE2.1 (latently infected), or HE2.1 treated with TPA for 18 hours (reactivation from latency). Northern blots were probed with strand-specific *M3/M2* locus probes B or C. Methylene blue staining is shown for rRNA as a loading control.

We have clearly noted the expression of *M3-04* in lytically infected fibroblasts. To examine whether this transcript is also expressed in latently infected cells or in cells reactivating from latency, we performed northern blotting on total RNA extracted from the MHV68 latently infected or reactivated B cell line HE2.1 (Figure 6). HE2.1 cells were previously generated via infection of the parental A20 mouse B cell lymphoma cell line with a hygromycin-resistant recombinant MHV68 [22]. While *M3-04* was clearly expressed during lytic infection (“WT” infection in the “3T12” probe B northern blot), this transcript was not detected during latency (“Ø” in the “HE2.1” probe B northern blot) or 18 hours after TPA induction of reactivation (“TPA” in the “HE2.1” probe B northern blot). In contrast, the transcript encoding M3 protein, *M3-01*, was highly expressed during both lytic infection and upon reactivation from latency (probe C northern blots), consistent with the abundant lytic expression of M3 protein. Likewise, *M3-01* was detected at a low level in latently infected cells, consistent with previous reports of low level M3 expression during latency. Together, these findings strongly suggest that *M3-04* is not expressed during latent infection; however, it is important to note that very low level *M3-04* expression could be below the threshold of detection in northern blots.

### 3.4. The M3-04 lncRNA Is Regulated by Antisense MicroRNAs

**Figure 7:**
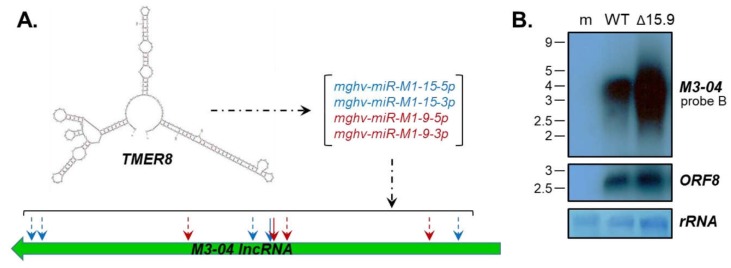
*M3-04* is regulated by *TMER8*-encoded microRNAs (miRNAs). (**A**) Diagram shows in silico (m-Fold) prediction of *TMER8* secondary structure, mature miRNAs encoded by *TMER8*, and position of *TMER8* miRNA binding within the *M3-04 lncRNA.* (**B**) Total RNA was harvested at 18 hours from mock-infected (m), wild-type MHV68-infected (WT), or MHV68∆15.9-infected (∆15.9) NIH 3T12 fibroblasts. Northern blots were probed with strand-specific *M3/M2* locus probe B or probe to the MHV68 early gene *ORF8*. Methylene blue staining is shown for rRNA as a loading control.

It is notable that the *M3-04* lncRNA is not only overlapping with the protein coding genes *M3* and *M2,* but also lies antisense to three miRNA-encoding *TMER* genes. Of particular interest is *TMER8,* which in addition to encoding a sequence fully complementary to the *M3-04* transcript, encodes three mature miRNAs that are predicted to target *M3-04* transcripts outside of the direct complementary sequence (Figure 7A, Table S2). Thus, we questioned whether the TMER8-encoded miRNAs *mghv-miR-M1-15-5p, -15-3p, -9-5p* and -*9-3p* may specifically regulate *M3-04* stability. To test this possibility, we infected fibroblasts with wild-type MHV68 or with MHV68Δ15.9, an MHV68 mutant lacking expression of pre-miRNAs *miR-M1-15* and *miR-M1-9*. Interestingly, in the absence of the *TMER8*-encoded miRNAs, we detected significantly increased levels of *M3-04* expression (Figure 7B). In contrast, expression of the control MHV68 transcript *ORF8* (Figure 7B) and surrounding genes *M1* and *M4* (Figure S1) was unchanged. Together these findings indicate that *TMER8*-derived miRNAs likely regulate the expression or stability of the *M3-04* lncRNA. 

### 3.5. Increased M3-04 Transcription In Vitro Correlates with Enhanced Lytic Replication In Vivo

**Figure 8:**
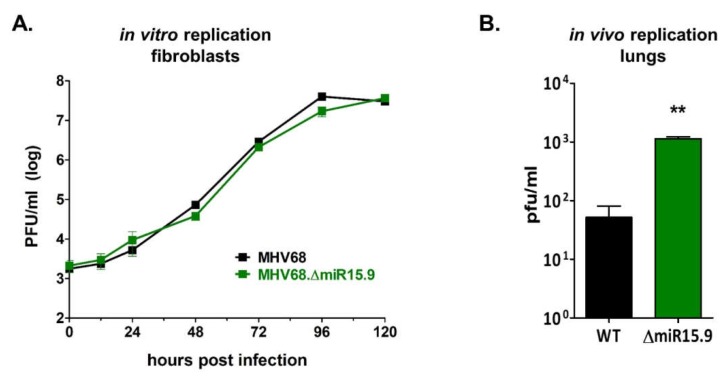
MHV68 mutant deficient in *pre-miRNA-15* and *pre-miRNA-9* replicates normally in vitro but demonstrates increased lytic replication in vivo. (**A**) Lytic replication in fibroblast cells in vitro was determined using multi-step growth curve. NIH 3T12 fibroblasts were infected with MHV68 or MHV68∆15.9 at MOI 0.05. Samples were then harvested at 12 to 24 hr increments and subjected to plaque assay to determine viral titer. Values represent the mean titer ± standard deviation (SD) (*n* = 3). (**B**) In vivo replication in lungs of wild-type mice was quantified by plaque assay. Eight mice per sample group were inoculated intranasally (i.n.) with 10^4^ PFU wild-type MHV68 (WT) or MHV68∆15.9 (∆15.9). At 5 dpi lungs were harvested and virus titers were determined by plaque assay. Values represent mean titer ± SD. ** *p* < 0.01 using an unpaired *t*-test.

Consistent with previous results demonstrating that the MHV68 miRNAs have no direct effect on lytic replication [23], mutation of MHV68 miRNAs *mghv-miR-M1-15* and *-9* has no direct effect on lytic replication in fibroblasts (Figure 8A). However, because the *M3-04* lncRNA directly overlaps the gene which encodes the critical latency protein M2, we questioned whether increased *M3-04* expression may alter the latent-lytic balance in favor of lytic replication in scenarios in which M2 may be expressed. To test this, we quantified titers of wild-type MHV68 versus MHV68Δ15.9 virus during the acute phase of infection in lungs in vivo, during which multiple cell types were infected and latent infection was beginning to be established. Wild-type C57BL/6J mice were intranasally inoculated with wild-type MHV68 or with the miRNA mutant virus MHV68Δ15.9. At 5 dpi, lungs were harvested and virus titers were quantified by plaque assay (Figure 8B). Notably, the mutant virus MHV68Δ15.9 displayed significantly increased lung titers in vivo compared to wild-type virus, a finding which is consistent with a potential role for the *M3-04* lncRNA in promoting lytic infection.

## 4. Discussion

In an effort to validate and examine the newly identified MHV68 transcripts, we performed detailed molecular analyses on transcripts within the important *M3/M2* latency locus. In so doing, we verified the presence of the novel 3.91 kb transcript *M3-04*, which overlaps both *M3* and *M2*, and likely arises from readthrough of the primary M3 coding transcript *M3-01*. We demonstrated that *M3-04* is polyadenylated and localized to the nucleus, strongly suggesting that this transcript is not translated and instead may function as a lncRNA. Consistent with a role for this putative lncRNA during lytic replication, (i) *M3-04* was consistently detected during lytic replication, but was not detected during latent infection or reactivation, and (ii) a virus mutation that leads to vastly increased *M3-04* expression also leads to significantly enhanced virus replication in vivo.

*M3/M2* is a fascinating locus and likely key control point for regulation of latent versus lytic infection. In addition to the highly expressed M3 chemokine binding protein, this region encodes the highly important B cell signaling protein M2, which is expressed solely in latently infected cells [24,25]. The region also harbors *TMER*-encoded miRNAs that are constitutively expressed and play important roles in infection and pathogenesis [23,26,27,28,29,30]. Intriguingly, similar control regions containing latency genes, miRNAs and antisense lncRNAs are present in EBV, KSHV, and HSV-1 genomes [31,32].

**Figure 9:**
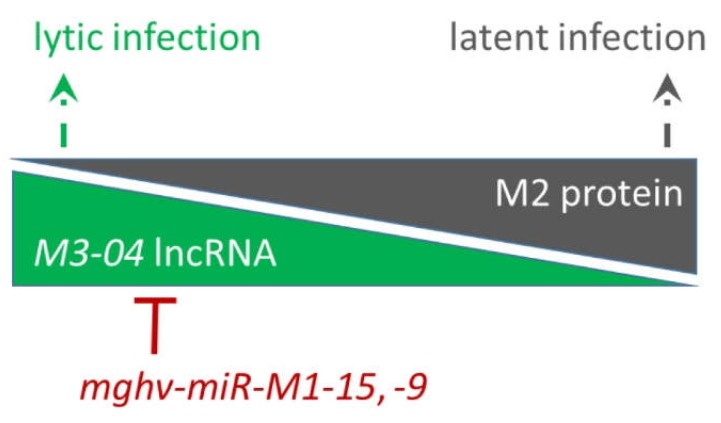
Working model for possible regulatory relationship between *M3-04 lncRNA*, *M2*, and antisense miRNAs *mghv-miR-M1-15 and -9*. During lytic infection, transcription of *M3-04* lncRNA may interfere with the expression of *M2* expression, facilitating lytic replication of the virus. In contrast, during latency, *M3-04* transcript is not expressed and the expression of M2 protein facilitates B cell signaling and the establishment of latency. Antisense miRNAs may regulate the level of the *M3-04* transcript in order to restrict lytic replication and/or to prevent leaky *M3-04* expression during latency establishment.

*M3-04* is the second-most abundant transcript expressed in this region, trailing only the highly expressed *M3-01* transcript, which encodes the M3 protein. That *M3-04* is detected during lytic replication in fibroblasts, but is not detected in latently infected or reactivated B cells, suggests that *M3-04* expression is dependent upon either state of infection or infected cell type. Nevertheless, the finding that *M3-04* is expressed during lytic replication, but the gene that it overlaps, the critical latency protein M2, is expressed only during latent infection suggests that the *M3-04* lncRNA may function to negatively regulate M2 expression during lytic replication (Figure 9). There is significant precedence for the possibility that *M3-04* transcription, rather than the lncRNA itself, may interfere with M2 expression. For example, in mammals, the *Igf2r* locus, which encodes insulin-like growth factor 2 receptor, is imprinted by the paternally expressed lncRNA *Airn*. *Airn* transcription directly overlaps with the *Igf2r* promoter and interferes with *Igf2r* expression [33]. Thus, it is conceivable that *M3-04* may function in a similar fashion during lytic infection to prevent M2 from initiating pro-latency signaling events in the infected host cell.

Within the critical *M3/M2* region of the genome, it would seem important to maintain a strict level of control over regulatory transcripts. As has been described for other viruses such as EBV and SV40 [34,35], such control could reasonably be accomplished through the expression of repressive antisense miRNAs. It is highly plausible that *TMER8*-encoded miRNAs could negatively regulate *M3-04* transcript levels. In addition to the specific complementarity of the antisense miRNAs themselves, there are at least 10 other locations within *M3-04* where TMER8-derived miRNAs are predicted to bind with high affinity. Our use of the MHV68Δ15.9 virus lacking *mghv-mir-M1-9* and *-15* revealed substantially increased levels of *M3-04* in the absence of these miRNAs, demonstrating a clear regulatory relationship between *M3-04* and these antisense miRNAs. Consistent with the possibility of miRNA targeting of a nuclear RNA, recent reports have emerged of miRNA targeting of nuclear transcripts involving Ago localization to the nucleus [36], including during KSHV infection [37]. Whether miRNA regulation of *M3-04* is mediated strictly through targeting at the *M3-04* complementary sequence, other target sequences within *M3-04*, or a combination of both remains unknown. However, we note that we do not detect any stable cleavage products, which can persist when transcript instability is directed strictly by complementary sequence-mediated cleavage events. It is also worth noting that miRNA regulation of *M3-04* stability may also play a role in our lack of *M3-04* detection during reactivation, as the abundant presence of miRNAs prior to reactivation may immediately destabilize any newly made *M3-04* transcripts. 

Notably, the *mghv-mir-M1-9* and *-15* mutant virus also displayed increased viral titer in the lung during acute infection, providing a strong correlation between increased levels of *M3-04* and enhanced lytic replication in vivo. Consistent with the possibility that *mghv-mir-M1-9* and *-15* may facilitate lytic replication indirectly through regulation of the *M3-04* lncRNA, mutation of *mghv-mir-M1-9* and *-15* did not alter lytic replication in NIH 3T12 fibroblasts in vitro, demonstrating that the effect of the miRNAs on lytic replication in vivo is indirect. Although the requirement for northern blot analysis for detection of specific transcript isoforms has thus far precluded determination of miRNA regulation of *M3-04* in in vivo samples, these findings together support the concept that *mghv-mir-M1-9* and *-15* may contribute to the control of the latent-lytic balance in the complex in vivo environment. Consistent with this possibility, the TMER8-derived miRNAs, and particularly *mghv-miR-M1-15-5p,* are abundantly expressed in lytically infected cells and are also expressed during latent infection in vivo [23]. However, at this time we cannot conclude whether the in vivo phenotype is a direct effect of increased levels of *M3-04*, or some other consequence of mutation of *TMER8* or the *TMER8*-encoded miRNAs. Future mechanistic work will need to be done to more precisely distinguish these possibilities.

The findings presented here validate the presence of a novel MHV68-encoded lncRNA and reveal an interesting regulatory relationship between multiple RNAs expressed within this region. Moreover, these observations emphasize that the MHV68 transcriptome is much more complex than previously appreciated, with multiple overlapping transcripts expressed from regions previously thought to be transcriptionally silent. This work also serves to underscore the point that studies of gammaherpesvirus genes within the context of the virus need to take into close consideration the possibly of transcripts generated from neighboring intergenic regions. These complex relationships much be considered in order to delineate whether specific phenotypes are associated with the gene of interest itself, or instead with overlapping or antisense regulatory transcripts. 

## Supplementary Materials

The following are available online at https://www.mdpi.com/2311-553X/5/1/6/s1, Table S1: sequences of primers used for northern blot probes and qRT-PCR, Table S2: TMER8 sequences of MHV68 and MHV68.Δ15.9, Figure S1: validation of mutant virus expression of *M1* and *M4*.
