# Supplementary material for: Gammaherpesvirus Readthrough Transcription Generates a Long Non-Coding RNA That Is Regulated by Antisense miRNAs and Correlates with Enhanced Lytic Replication In Vivo
Source: Noncoding RNA. 2019 Jan 10;5(1):6. doi: 10.3390/ncrna5010006 (PMC6468771; doi:10.3390/ncrna5010006)
Supplement: Supplementary file 1 [file ncrna-05-00006-s001.pdf]

Supplementary Materials

Gammaherpesvirus readthrough transcription generates a long noncoding RNA that is regulated by antisense miRNAs and correlates with enhanced lytic replication in vivo

Mehmet Kara, Tina O'Grady, Emily R. Feldman, April Feswick, Yiping Wang, Erik K. Flemington and Scott A. Tibbetts *

Table S1. Sequences of primers used for northern blot probes and qRT-PCR.

Primer	Sequence
TMER4 T7 FWD	TAATACGACTCACTATAGGGAGA CCCATCACCCCGTCATAAGAG
TMER4 Sp6 REV	ATTTAGGTGACACTATAGAAGTG CATCGCAAGCGTTTCCACC
M3 probe A T7 FWD	TAATACGACTCACTATAGGGAGA GAGGGACGAGATTGTGCTAG
M3 probe A REV	ATTTGCATGTGTGTGAGCAG
M3 probe B T7 FWD	TAATACGACTCACTATAGGGAGA CAGTTGGAACCTCCAGTCTCG
M3 probe B REV	GAGTCCCACCACACAGTTTG
M3 probe C T7 FWD	TAATACGACTCACTATAGGGAGA ATGCCAGCACACTCAGTCAC
M3 probe C REV	AGAAAGTGTTGTGTTGAGGT
ORF8 FWD	CACGCTCAGTAGACCTGGACAC
ORF8 Sp6 REV	ATTTAGGTGACACTATAGAAGTG GCCCTCCACATGCTTCTTATC
Malat1 FWD	GGGAGTTGTAGGCTTCTGTG
Malat1 T7 REV	TAATACGACTCACTATAGGGAGA TCTGGTTCCCTTGAGTCATC
M1 FWD	TTTGAAAGTAGACTCCCACTACCCA
M1 REV	TTAGAGTATGTAACCATGGCCAGGT
M4 FWD	ATTCTATTGGGCAATCAGGGAT
M4 REV	AAAGAAACCAAAGGACGCAG
GAPDH FWD	CATGGCCTTCCGTGTTCCTA
GAPDH REV	CCTGCTTCACCACCTTCTTGAT

Table S2. TMER8 sequences of MHV68 and MHV68.Δ15.9.

Virus	TMER8 Sequence
MHV68	GCACCAGAGTGGCTCACCTGGTAGAGCACCAGGCTGCCCATCCTGTTGGTTCTC<br>GGTTCAAATCCGAGCTCTGGTGACT**AGCTACCCGCGTGGCCGGAGTGTTTAGAC**<br>**CCTCTAACCCACCTTCCGGGTCTCTCCTTTATAATTGAGGTTCCCGGCAAATGTT**<br>**GGATAGATAGGTAACTTCTCACATTTGCCTGGACC**TTTTTTGCC
MHV68.<br>Δ15.9	GCACCAGAGTGGCTCACCTGGTAGAGCACCAGGCTGCCCATCCTGTTGGTTCTC<br>GGTTCAAATCCGAGCTCTGGTGACTTTTTTGCC

Boxes indicate the locations of pol III promoter box A and box B. Bold indicates the sequence deleted in the mutant virus. Underlines indicate the position of mghv-miR-M1-15-5p, -15-3p, -9-5p, and -9-3p, respectively.

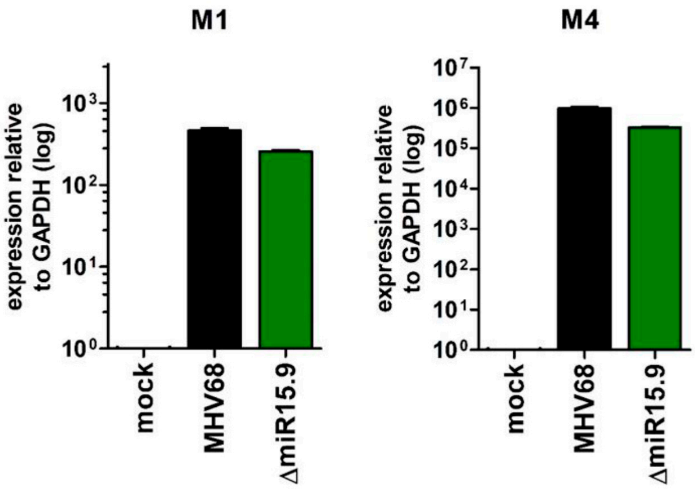

Figure S1. Validation of mutant virus expression of *M1* and *M4*. Following 18 hr infection of NIH 3T12 fibroblasts with mock, wild-type MHV68, or MHV68.ΔmiR15.9, expression levels of adjacent genes *M1* and *M4* were verified by qRT-PCR. Expression levels of *M1* and *M4* are presented as relative to *GAPDH* housekeeping control.
